# Cancer Cell Dormancy and Chemotherapy Resistance

**DOI:** 10.7150/jca.123742

**Published:** 2026-01-01

**Authors:** Jingxian Hu, Wei Zhou, Ying Zhao, Peiran Li, Zhenying Zhao, Wei Wang, Wenhong Wang, Yijia Wang

**Affiliations:** 1 Tianjin University of Traditional Chinese Medicine, Tianjin, 301617, China.; 2 Department of Neurology, Tianjin Union Medical Center, The First Affiliated Hospital of Nankai University, Tianjin, 300121, China.; 3 Human Biology and Society B.S., University of California, Los Angeles, 90095, America.; 4 Department of Pharmacy, Tianjin Union Medical Center, The First Affiliated Hospital of Nankai University, Tianjin, 300121, China.; 5 TEDA Institute of Biological Sciences and Biotechnology, Nankai University, Tianjin, 300457, China.; 6 Department of Radiology, Tianjin Union Medical Center, The First Affiliated Hospital of Nankai University, Tianjin, 300121, China.; 7 Tianjin Institute of Coloproctology, Tianjin Union Medical Center, The First Affiliated Hospital of Nankai University, Tianjin, 300121, China.

**Keywords:** chemotherapy, dormancy, immunity, hypoxia, metabolism

## Abstract

Cancer cell dormancy is associated with tumor recurrence and metastasis. Chemotherapy usually induces dormancy as external pressure on the tumor. Dormant cells have considerable resistance to antitumor drugs, although they are not harmful to the host if they do not wake up. Chemotherapy induces a dormant phenotype through remodeling of the tumor microenvironment and alteration of intracellular signaling networks. Multiple adaptive mechanisms that confer drug resistance have been identified in these dormant cells, including the unfolded protein response to endoplasmic reticulum stress, metabolic reprogramming favoring oxidative phosphorylation to avoid damage from oxidative stress, and autophagy to realize the circular utilization of energy. However, dormancy is reversible. The conversion between dormancy and awakening of the tumor during chemotherapy and the recovery period after treatment is modulated by several factors, including the dose and cycle of treatment, and individual differences among patients. The direct elimination of cancer cells or permanent dormancy by chemotherapy predicts favorable outcomes. According to this theory, understanding the mechanisms of cancer dormancy and awakening under chemotherapy and improving prognosis using suitable treatment strategies requires further investigation. This review analyzed studies on cancer cell dormancy and response to chemotherapy to identify potential novel interests for future studies and probable strategies to optimize chemotherapy in clinical trials.

## Introduction

Chemotherapy is among the primary treatments for cancer; however, it often fails to eradicate all tumor cells. A subset of cancer cells are capable of maintaining dormancy both locally and at distant sites—termed minimal residual disease (MRD)—and may persist under therapeutic pressure for years or even decades 1. Some perspectives suggest that MRD—namely, disseminated tumor cells (DTCs)—may persist in a dormant state and ultimately cause metastatic disease 2. More than 90% of cancer-associated mortality is due to metastatic disease 3. Therefore, to improve patient prognosis, it is essential to understand how DTCs (MRD), evade treatment and persist long-term. One prevailing hypothesis is that DTCs evade elimination by adopting a dormant state 4.

Dormant cancer cells are defined as non-proliferating cancer cells that undergo G0 or G1 cell cycle arrest 5. Treatment stress induces a reversible shift to dormancy as an adaptive survival strategy, rather than selecting pre-existing resistant clones 6. Dormant cells survive therapy by arresting the cell cycle, making them insensitive to treatments targeting proliferating cells 7. Apoptosis in cancer cells is a common mechanism underlying the activity of chemotherapeutic agents 8. However, chemotherapy can induce either dormancy or reactivation, depending on dose and schedule. In brain-metastatic breast cancer spheroids, low-dose paclitaxel (< 80 nM) promotes dormancy via reduced p-ERK/p-p38 signaling, whereas higher doses (> 200 nM) trigger apoptosis 9. An independent study shows that docetaxel (0.01-10 μM *in vitro*; 8 mg/kg *in vivo*) is another plant alkaloid chemotherapeutic agent, which can damage stromal cells in the tumor microenvironment (TME), resulting in the release of tumor promoting cytokines from these cells. These factors activate dormant cancer cells and promote their proliferation *in vitro* and *in vivo* via the MEK/ERK pathway 10.

Chemotherapy shows dual effects: while it can effectively suppress tumor growth, it may paradoxically contribute to tumor progression, potentially by inducing cellular dormancy or reactivating dormant cancer cells 11-13. The regulation of dormancy by drugs should be considered when designing treatment strategies because dormancy can significantly affect treatment outcomes. A deeper understanding of how chemotherapy induces dormancy and how dormant cancer cells adapt to and survive treatment stress is critical for optimizing drug strategies and preventing relapse. This review discusses the complex interrelationships between chemotherapy and tumor cell dormancy to provide insights into chemotherapy.

## 1. TME

**Figure 1:**
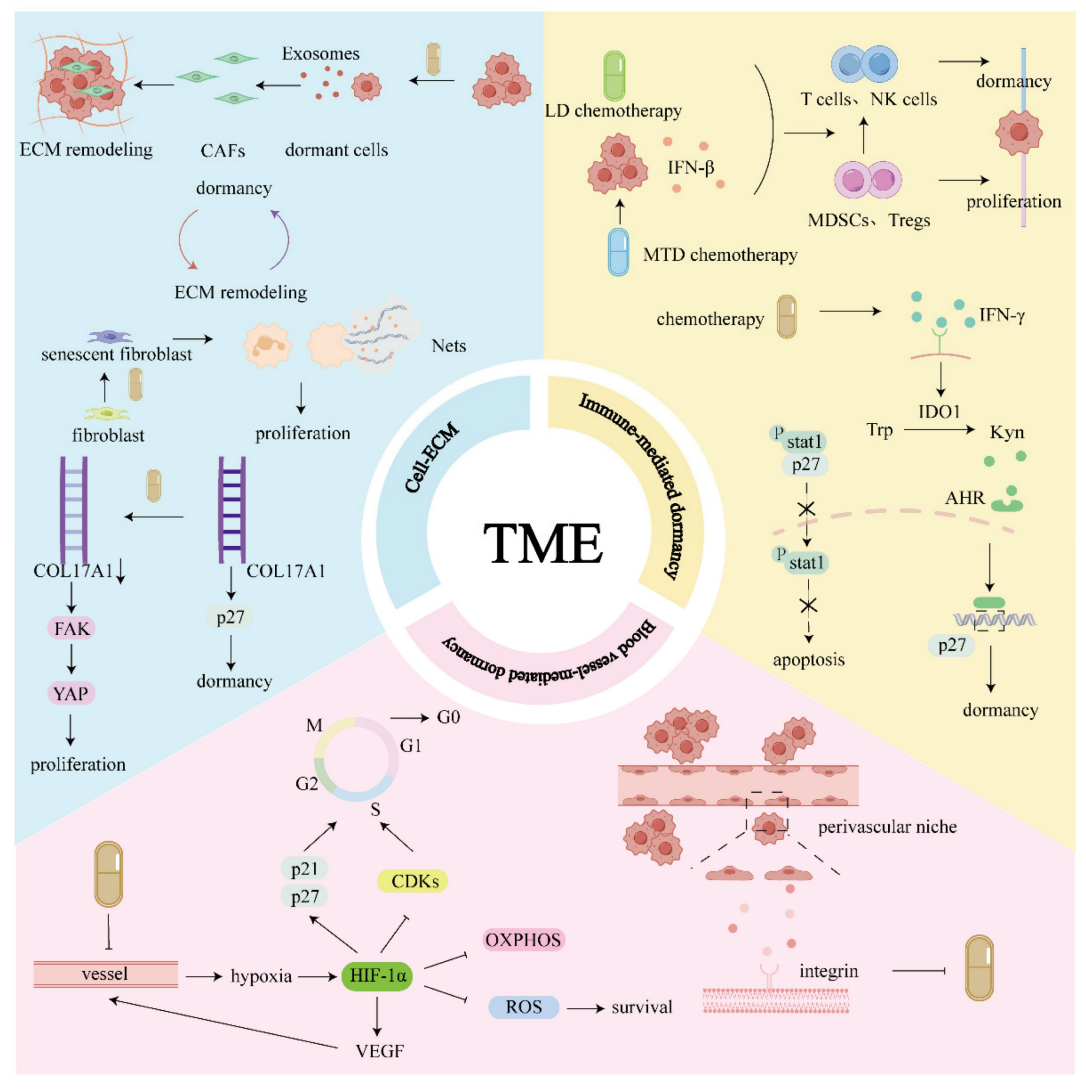
TME: Immune cells are directly involved in dormancy. High-dose and low-dose chemotherapy can promote the transition from the microenvironment dominated by immunosuppressive cells to the microenvironment dominated by anti-tumor immune cells, promoting dormancy. At the same time, chemotherapy can induce IFN-γ secretion, which makes TRCs tend to be dormant rather than apoptotic. Chemotherapy can induce the expression of hypoxia-inducible factor HIF-1α by damaging blood vessels, leading to tissue hypoxia. HIF-1α not only promotes tumor cells entering a dormant state but also helps to maintain the survival of dormant cells. Additionally, DTCs can acquire resistance to chemotherapy in the perivascular niche, and this resistance is partially attributed to protective factors secreted by endothelial cells, as well as regulatory mechanisms associated with integrin signaling pathways. Chemotherapy promotes cells to enter/exit dormancy, and dormant cells will remodel ECM. Chemotherapy promotes ECM remodeling and further affects dormant cells.

Elements of the TME include blood vessels, extracellular matrix (ECM) components, and non-malignant stromal cells 14. The cellular and noncellular components of the TME are essential for the induction and maintenance of tumor cell dormancy (**Fig. 1**).

### 1.1 Immune-mediated dormancy

The immune system plays a dual role in cancer progression, either suppressing tumors or promoting their development. To capture this paradoxical process, the concept of “cancer immunoediting” was introduced 15. Cancer immunoediting consists of three phases: elimination, equilibrium, and escape. Immune-mediated dormancy occurs during equilibrium phase 16. Immune-mediated dormancy can be considered as the equilibrium phase, as both reflect a dynamic balance between the immune system's anti-tumor and pro-tumor activities 17.

The equilibrium phase lasts for a long time, during which the immune system and tumors sustain a dynamic balance, maintaining tumor cells in a dormant state. Nevertheless, the interaction is based on the existence of cancer-cell immunogenicity and immune surveillance. When immunogenicity or immune function declines, dormancy may be disrupted, leading to immune escape 18. Clinically, tumors often form masses after successfully evading immune surveillance; therefore, most malignant tumors are in the immune escape phase at diagnosis 16. Immune evasion may occur even in the presence of tumor immunogenicity and host immune functions. The study found that dormant DTCs express lower levels of major histocompatibility complex class I, yet the molecules remain functionally active, indicating that the immune evasion of DTCs was not caused by the lack of immunogenicity. This is because both DTCs and endogenous antigen-specific T cells are relatively rare populations, indicating that the interactions between them are also rare. Therefore, therapeutic strategies designed to increase the probability of such interactions may help clarify dormant DTCs, thus reducing the likelihood of recurrence and improving prognosis 19.

The immune system is directly involved in maintaining dormancy in cancer cells 20. For their role in maintaining dormancy, immune cells are broadly classified into two groups. The first comprises immune effector cells that promote and sustain dormancy, including effector T cells and natural killer (NK) cells. Notably, the duration of tumor cell dormancy is closely tied to the presence of CD8⁺ T cells. Depletion or exhaustion of CD8⁺ T cells has been shown to accelerate metastatic progression or shorten the time to recurrence, highlighting their critical role in maintaining dormancy. When these cells become exhausted, dormancy may be reversed 21, 22. Clinical and experimental evidence indicates that dormant DTCs reside in the bone marrow, where CD4+ and CD8+ T cells levels are elevated 23, 24. Mice with dormant sarcomas showed an increased proportion of NK cells compared with mice with advanced sarcomas 25. The second group consists of immunosuppressive cells that facilitate the reactivation of dormant tumor cells, including regulatory T cells (Tregs) and myeloid-derived suppressor cells (MDSCs). Both Tregs and MDSCs are known to promote tumor progression by suppressing anti-tumor immune responses. In various tumor models, Treg infiltration exhibits heterogeneity—dormant tumors may show either decreased or increased Treg levels compared to progressive tumors. However, Tregs consistently retain their ability to suppress both CD4⁺ and CD8⁺ T cell activity, regardless of their abundance 25, 26. MDSCs are thought to reverse dormancy of DTCs 27. MDSCs suppress anti-tumor immunity both directly, by inhibiting T cell responses, and indirectly, by promoting the expansion of Tregs 28. The interactions described above are largely indirect; direct evidence linking dormant DTCs and MDSCs remains limited and warrants further investigation.

Chemotherapy modulates the host immune system. Chemotherapeutic agents such as doxorubicin (DOX), oxaliplatin (OXA), and camptothecin can activate the immune response through immunogenic cell death 29. Immunogenic cell death enhances tumor immunogenicity through the release of damage-associated molecular patterns and surface exposure of immunostimulatory signals. This process activates both innate and adaptive immunity, recruits dendritic cells, and elicits cytotoxic T cell responses, ultimately promoting tumor suppression and durable immune memory.^29^. Therefore, chemotherapy can regulate cell dormancy via the immune system.

Chemotherapy can induce dormancy through immune modulation. In 4T1 mammary tumors, high-dose DOX (anthracycline) and methotrexate (antimetabolic) activate the IRF7/interferon (IFN)-β/IFNAR axis, shifting the immune response from MDSCs-dominated to CD4+/CD8+ T cell-dependent anti-tumor response that maintains dormancy 30. Modeling studies further show that such dormant, IFN-active cells may serve as prophylactic or therapeutic vaccines to suppress aggressive breast cancer progression, offering a potential immunotherapeutic strategy when combined with chemotherapy 31. The chemotherapy concentrations used in this study simulated the maximum tolerated dose (MTD) chemotherapy. MTD chemotherapy promotes immunity by producing IFN-β 30. Although MTD chemotherapy can inhibit tumor growth, MTD chemotherapy is generally believed to impair the immune system, including lymphopenia, myelosuppression, severe weight loss, and a reduction in CD8+ T cells 32. Therefore, chemotherapy may reduce the number of immune cells; nevertheless, it may also indirectly enhance antitumor immunity via immunophenotypic shifts and activation of type I IFN signaling. The relationship between chemotherapy and immune response is complex. They can either promote or suppress immune responses. MTD chemotherapy is a short-term high-dose regimen, the impact of long-term low-dose chemotherapy, such as metronomic chemotherapy and adaptive therapy, on tumors and the immune system needs to be investigated. Low-dose metronomic chemotherapy can also promote a switch in immune response from immunosuppression to antitumor immunity. Metronomic chemotherapy is a new method of chemotherapy that refers to the frequent low-dose administration of conventional chemotherapy drugs without prolonged drug-free breaks 33. The standard clinical protocol for chemotherapy generally uses the maximum drug dose that the patient can tolerate, which requires prolonged intervals between treatment cycles for normal tissue to recover from the cytotoxic effects of the drug 34.

The oral administration of metronomic cyclophosphamide selectively depletes Treg cells and activates peripheral T cells and NK effectors in patients with advanced cancer 35. Metronomic capecitabine also reduces MDSCs in preclinical models 36. Low-dose metronomic chemotherapies, including PTX, DOX, and cisplatin (CDDP), can augment CD8⁺ T cell-mediated cytotoxicity via endogenous type I IFN produced by cancer cells 32. Immune cells are directly involved in maintaining the dormant state of cancer cells. To a certain extent, maintenance of dormancy in DTCs depends on the immune surveillance system, especially tumor-infiltrating lymphocytes and NK cells 37. When MTD and low-dose chemotherapy promote tumor-infiltrating lymphocytes and NK cells, and inhibit Tregs and MDSCs, chemotherapy can help maintain cell dormancy in some cases.

However, inappropriate low-dose chemotherapy dosages and treatment regimens may result in the opposite outcomes. Low-dose chemotherapy can also promote tumor growth, and gemcitabine alone or gemcitabine combined with CDDP, and half of the metronomic dosages can significantly promote tumor formation and growth 38. Improper low-dose chemotherapy regimens activate dormant microtumors, thereby promoting tumor recurrence and metastasis. The optimal dose and schedule of low-dose chemotherapy, including metronomic chemotherapy, remain largely empirical. Therefore, further investigation is required to refine this therapeutic method and avoid potential risks 38. Maintaining cells in a dormant state can prevent recurrence for a period; however, completely eliminating dormant cells ensure no risk of relapse.

As a type II IFN, IFN-γ is involved in the mechanism by which chemotherapy promotes apoptosis and dormancy. Chemotherapy induces IFN-γ production in various cell types 39. Different drugs and treatment combinations can have different effects on IFN-γ. For example, CDDP can promote IFN-γ production 40. CDDP combined with the plasmid encoding interleukin-12 gene can also activate immune effector cells to release IFN-γ 41. However, adding pembrolizumab to docetaxel and cisplatin (TP) (i.e., pembrolizumab + TP) significantly inhibited IFN-γ 42. IFN-γ secreted by T cells can induce stem-like bladder cancer cells to enter dormancy via IDO/Kyn/AHR/P27 cascade reaction 43. When activating the IDO/Kyn/AHR/P27 cascade by IFN-γ in tumor-repopulating cells (TRCs), this pathway can induce dormancy, although IFN-γ can also induce apoptosis by activating caspase 3 and 7 via JAK/STAT1. When IDO1 or AhR inhibitor is used in blocking IDO/Kyn/AHR/P27 cascade, IFN-γ tends to direct TRCs toward the JAK/STAT1 pathway. Therefore, the study proposes a novel therapeutic strategy: IFN-γ combined with IDO1 or AhR inhibitor can disrupt the dormancy of TRCs and induce their apoptosis, thus achieving more effective tumor clearance 44. A strategy that completely eliminates dormant cancer cells is better than maintaining tumor dormancy for a long time.

### 1.2 Blood vessel-mediated dormancy

The hypoxic microenvironment of the primary tumor is the reason why DTCs cells acquire cellular drug resistance. Experiments have proved that when DTCs experience a hypoxic microenvironment in the primary tumor, their dormant key factors and hypoxia markers will be synchronously upregulated, prompting DTCs to enter a dormant state. However, after DTCs metastasize to distal organs, they are still dormant phenotype, although they no longer maintain high expression of hypoxia markers. Further studies have found that such DTCs experiencing hypoxia are more likely to activate nuclear receptor subfamily 2 group F member 1 gene (NR2F1)-dependent dormant programs, thereby evading chemotherapy-induced apoptosis, suggesting that hypoxia within the primary tumor may be an important cause of treatment tolerance and recurrence 45.

Hypoxia prompts cells to enter dormancy 46, thus, it shows resistance to chemotherapy, which may be related to hypoxia-inducible factor-1α (HIF-1α). HIFs are primary transcription factors that respond to hypoxia 47, 48. For example, HIF-1α in platinum-resistant tissues is highly expressed and is extremely important for platinum resistance 49. Acquired drug resistance is linked to HIF-1α, which drives entry into a dormant state and maintains dormant-cell viability. As a hypoxia response regulatory factor, HIF-1α plays a key role in modulating cell dormancy, and HIF-1α can induce cells to enter a dormant state 50-52. HIF-1α directly causes cancer cells to a resting stage.HIF-1α induces G0/G1 cell-cycle arrest by upregulating p21 and p27^53^, and inhibition of cyclin-dependent kinases (CDKs) 54. In regulating metabolism, HIF-1α increases glucose transporter type 1 to enhance glucose intake and stimulates the expression of glycolytic enzymes to induce glycolysis, which both facilitate energy acquisition and survival in hypoxia microenvironment 55. HIF-1α inhibits oxidative phosphorylation (OXPHOS) 56 and reduces reactive oxygen species (ROS) production 57, thereby preventing apoptosis triggered by DNA damage and maintaining long-term survival of dormant cells. However, hypoxia does not always induce HIF-1α expression. For example, mild hypoxia in periphery region of large tumor tissue promotes HIF-1α. However, in peri-necrotic area where hypoxia is extreme, HIF-1α is downregulated, and the hypoxia-inducible gene domain family member 1A is increased. The latter represses respiration and total ROS, but induces AMP-activated protein kinase (AMPK) activity to promote survival 58. The regulatory effect of HIF-1α on metabolism may affect the prognosis of chemotherapy by promoting dormancy. HIF-1α-mediated inhibition of OXPHOS and ROS production plays a key role for dormant chronic myelogenous leukemia stem cells to survive and maintain potential for recurrence during tyrosine kinase inhibitors treatment 59. HIF-1α also activates vascular endothelial growth factor (VEGF) 60. VEGF is a specific mitogen for endothelial cells. Activation of VEGF promotes angiogenesis, potentially disrupting dormancy and promoting tumor growth 61. The HIF-1α/VEGF axis is identified as a key regulator of angiogenesis in many malignant tumors. For example, inhibition of HIF-1α degradation enhances VEGF secretion, thereby promoting tumor angiogenesis in colorectal cancer (CRC) 62. Targeting HIF-1α/VEGF mediated tumor angiogenesis can inhibit tumor progression 61. Therefore, when tumor angiogenesis increases, tumor cells may obtain oxygen and nutrients to exit dormancy and re-enter a proliferative state. However, metronomic chemotherapy-induced hypoxia upregulates HIF-1α, thereby activating downstream VEGF and enhancing angiogenesis, which merely alleviated this hypoxia state rather than reversing it completely.

In general, as a response to hypoxia, HIF-1α upregulation facilitates tumor survival. HIF-1α promotes dormancy under hypoxia and facilitates tumor angiogenesis to increase supply of oxygen. Many reports have indicated that HIF-1α contributes to chemoresistance 63, 64; thus, dormancy may be involved in the mechanism. In particular, metronomic chemotherapy targets vascular endothelial cells to inhibit angiogenesis, resulting in hypoxia, which promotes HIF-1α expression. HIF-1α promotes cellular dormancy and helps cells survive stress. However, HIF-1α also activates downstream VEGF, which drives angiogenesis, alleviates hypoxia, and may supply nutrients to cells, thus disrupting dormancy. In summary, the regulatory effects of anti-tumor drugs on HIF-1α can affect tumor dormancy and awakening during therapy, and individual differences of HIF-1α need to be considered when chemotherapy drugs were selected.

It is generally believed that DTCs exhibit resistance to chemotherapy due to their state of dormancy, i.e., in a state of cell cycle arrest. However, studies have found that DTCs can colonize the perivascular niche of the bone marrow for a long time, where they show remarkable resistance to conventional cytotoxic chemotherapy. This resistance is not dependent on the dormant state of DTCs, but is protected by vascular endothelial cells through integrin-mediated adhesion signals. Studies have further shown that endothelial-derived factors such as von Willebrand factor and Vascular Cell Adhesion Molecule-1 enhance DTCs' resistance to chemotherapy by activating integrinαvβ3 and α4β1 respectively. Inhibition of these integrins or their ligands by functional blocking antibodies or shRNA significantly increases the sensitivity of DTCs to chemotherapeutic drugs and effectively reduces the incidence of bone metastasis in mouse models. More importantly, this integrin blockade does not induce DTCs to exit their dormant state into the cell cycle, and no increase in chemotherapy-related toxicity is observed. These results suggest that the addition of integrin inhibitors before adjuvant chemotherapy may be a viable strategy to eradicate DTCs in micrometastatic lesions, thus lowering the likelihood of distant metastasis and recurrence 65.

### 1.3 Cell-ECM

Chemotherapy-induced dormant cells can remodel the ECM to adapt to chemotherapeutic stress, whereas chemotherapy can remodel the ECM to affect dormant cells.

Dormant lung adenocarcinoma cells are induced after 4.5 μg/mL CDDP. Exosomes derived from dormant cells activate cancer-associated fibroblasts (CAFs), leading to ECM remodeling 66. CDDP-induced dormant lung cancer cells (2 days, 10 ng/μL) promoted ECM remodeling by regulating ECM-related genes and signaling pathways. This process may function as a key mechanism for adaptation to chemotherapeutic stress and may eventually lead to cancer recurrence 67. However, chemotherapy may also potentially awaken dormant DTCs by inducing remodeling of the tumor microenvironment, promoting their re-proliferation and inducing metastasis. Chemotherapy can induce lung fibroblasts to enter a state of cellular senescence and secrete various typical senescence-associated secretory phenotype (SASP) factors, including IL-6, IL-8, and matrix metalloproteinases. These factors stimulate neutrophils to release neutrophil extracellular traps (NETs), which contain proteases. These proteases degrade ECM components such as laminin, thus releasing biologically active fragments. These fragments can activate the integrin signaling pathway on the surface of dormant DTCs, which, in turn, triggers ERK activation while suppressing p38 signaling, ultimately disrupting dormancy and promoting DTCs proliferation, potentially leading to cancer recurrence 12. Therefore, this also provides some new treatment ideas, such as the combined use of NETs inhibitors, targeting ECM remodeling or integrin pathways, and intervening in fibroblast senescence and SASP secretion. Similarly, NETs critically contribute to dormant cancer-cell reawakening. The proteases rich in NETs can degrade laminin-111 in the extracellular matrix, exposing specific epitopes, which are then recognized by the integrin α3β1 on the surface of dormant cancer cells, thereby activating downstream signaling pathways such as FAK/ERK/MLCK/YAP, prompting cancer cells to transition from dormancy to proliferation. It is noteworthy that this activation process relies not only on the enzymatic activity of NETs-related proteases but also on their DNA scaffold structure, which provides a spatial platform that enhances the co-localization of the enzyme and substrate, thereby increasing the hydrolysis efficiency of laminin. Based on this mechanism, inhibiting the formation of NETs or blocking the interaction of the remodeled laminin by NETs has become a potential therapeutic strategy 68.

Chemotherapy can affect dormant cells by inducing ECM remodeling. *In vivo* treatments, including the FOLFOXIRI regimen or irinotecan, and *in vitro* treatment comprising 5-Fluorouracil (5-FU), OXA, and camptothecin can promote the hydrolysis of type XVII collagen (COL17A1) (a cell-adhesion molecule that strengthens hemidesmosomes) and activate the FAK-YAP pathway, thereby prompting dormant LGR5p27 cancer cells to re-enter the cell cycle. Thus, the cell-matrix interface is a critical factor for maintaining the dormant state of cancer cells 11. The complex relationship between chemotherapy and the ECM should be elucidated. Because the ECM is multifunctional and dynamic, chemotherapy can change its composition, organization, and stiffness. These changes may promote the survival and awakening of dormant cells, ultimately triggering cancer recurrence and adversely affecting patient prognosis 69.

## 2. Internal Cellular Factors

### 2.1 P38 MAPK signaling pathway

The MAPK pathway is central to dormancy regulation 70. The MAPK pathway operates as a three-tier kinase module. The upstream kinase (MAPKKK) responds to a range of extracellular and intracellular signals by phosphorylating the intermediate kinase (MAPKK), thereby activating MAPK 71. MAPK is generally classified into mitogen-activated and stress-activated types, the classic representatives of which are extracellular signal-regulated kinase (ERK), c-Jun N-terminal kinase, and p38 (stress reactivity). From a physiological perspective, these kinases are not strictly classified, and all three families produce broad and overlapping responses to multiple signals 71. The ERK1/2 to p38 ratio can be used as an indicator of whether cancer cells are in a dormant state. When the p-ERK1/2 to p-p38 ratio is high, cancer cells are more inclined to proliferate. By contrast, when the ratio is low, cancer cells are more likely to enter dormancy 72.

Chemotherapy triggers cancer cells to enter dormancy by activating the p38 MAPK pathway. For example, chemotherapeutic agents such as DOX (5 mg/kg), PTX (10 mg/kg), and CDDP (5 mg/kg), for 48 h can promote the secretion of PTEN-long. PTEN-long induces a dormant phenotype in PTEN-deficient tumor cells via activation of the p38 MAPK pathway 73. Stress-induced p38 MAPK activation represents a key mechanism associated with tumor cell dormancy. Evidence indicates that a p38-regulated transcription factor network is essential for maintaining the dormancy of human squamous carcinoma cells. Activation of p38 elevates p53 and BHLHB3 and simultaneously suppresses c-Jun and FoxM1. Notably, the p38-driven induction of p53 specifically requires c-Jun downregulation. Downregulation of BHLHB3 or p53 disrupts dormancy, whereas depletion of c-Jun or FoxM1 promotes entry into a dormant state 74.

Cell cycle is driven by cyclins, CDKs, and CDK inhibitors (CKIs) 75. Dormancy or proliferative signals are ultimately converged on the regulation of CDKs. Once CDKs activity reaches the required threshold, cells enter the cell cycle and proliferate; otherwise, they remain dormant 76. Cell dormancy is commonly achieved by reducing cyclin-CDK activity, including the upregulation of CKIs or the degradation or downregulation of cyclins 76. CKIs comprise two families: the CIP/KIP family (p21Cip1/WAF1, p27Kip1, p57Kip2) 77 and the INK4 family (p15INK4b, p16INK4a, p18INK4c, p19INK4d) 78. Therefore, the p38 MAPK pathway eventually converges with cell cycle regulators. For example, after activation, p38 MAPK can phosphorylate the N-terminus of RB at pS249/pT252, thereby promoting the p27 expression, inhibiting cell proliferation, and promoting cells to enter the dormant state 79. P-p38α phosphorylates cyclin D, leading to ubiquitination and degradation of cyclin D 80. P38 MAPK triggers CKIs expression and induces cells to enter dormancy, which may be related to activation of the NR2F1 by p38 MAPK. 81. NR2F1 is the main regulator and activator of tumor cell dormancy 82. NR2F1 induces cell dormancy via SOX9 and NANOG. Additionally, SOX9 upregulates the expression of p16 and p27 83. NANOG, a transcription factor 84, upregulates the transcription of p21 and p27, thereby promoting cell dormancy 85.

### 2.2 P53

Genotoxic chemotherapeutics can damage DNA, thereby activating the DNA damage response, a conserved mechanism involving a wide range of proteins that collaborate to repair damaged DNA and determine cell fate 86. P53 protein plays a pivotal role in the DNA damage response 87.

As an essential stress-response mechanism, the p53 pathway integrates diverse upstream signals and actives multiple downstream pathways. Among these, cell cycle arrest and apoptosis are the most well-known pathways; p53 is also involved in the regulation of cell senescence 88. The p53 pathway, once activated, drives outcomes that span transient responses (e.g., DNA repair, cell-cycle arrest), terminal fates (e.g., apoptosis), and permanent cell-cycle exit (senescence) 89.

On the one hand, activation of p53 causes cell dormancy and can also cause cell proliferation. P53 can induce p21 production, thus mediating dormancy. Evidence indicates that the increased tolerance to DNA damage in a subset of cells is mechanistically attributable to ATM/ATR-p53-p21-driven cellular dormancy 90. After exposure to DNA-damaging agents, cancer cells arrest the cell cycle via the p53-p21 axis as a survival strategy 91 92. A complex regulatory relationship exists between p21 and p53. Normal tissue expression of p21 does not depend on p53, whereas the expression of p21 induced by DNA damage agents depends on p53 93. In addition, the tumor-suppressive effect of p21 may be transformed into an oncogenic function under certain circumstances, especially when p53 is mutated or when p53 levels are low, resulting in p21 levels also being low 94. For example, Gain-of-function mutant p53 transcriptionally upregulates p21 expression, and p21, in turn, promotes proliferation via a non-canonical mechanism 95. In addition, p53 induces G2/M phase arrest by inhibiting CDKs and cyclin B 96.

By contrast, p53 promoted apoptosis. When cells are damaged by genotoxicity, p53 is activated and stabilized. P53 then binds to and activates the promoter regions of its downstream genes, particularly those containing the p53 response element. These downstream genes include classical pro-apoptotic genes, such as BCL-2 family members (PUMA and BAX), and non-classical pro-apoptotic genes, such as p53 related to PMP-22. By activating these genes, p53 promotes apoptosis 97, 98. Under normal circumstances, newly synthesized p53 accumulates in the nucleus, and only a small amount is present in the cytoplasm, where p53 is strictly modulated by ubiquitination and proteasomal degradation. In the presence of apoptotic stressors, such as chemotherapy, numerous p53 proteins accumulate in the cytoplasm and promote apoptosis by directly targeting the mitochondria. For example, when hepatocyte odd protein shuttling inhibits degradation of the cytoplasmic p53 proteasome to stabilize p53 expression, p53-dependent mitochondrial apoptosis will be triggered 99. Inactivation of the p53-mediated apoptotic pathway can lead to chemotherapy resistance. One study has demonstrated that chemotherapy induces ERK to phosphorylate radical fringe Ser255 residues. Phosphorylated radical fringe S255 inhibits p53 phosphorylation, ultimately suppressing both apoptosis and ferroptosis, thereby mediating OXA resistance in CRC 100.

The activation of p53 can modulate cellular senescence. In most cancers with wild-type p53, p21 preferentially promotes premature senescence under stress rather than apoptosis. Senescence-associated β-galactosidase is a common marker of senescent cells. However, the existence of senescence-associated β-galactosidase alone does not rule out the possibility that the cells are in a quiescent state or undergoing differentiation, as these processes are gradual with transitional phases 101. In chemotherapy-exposed breast cancer, wild-type p53 tends to induce senescence, not apoptosis. Senescent cells can persist and cause residual disease and, through the SASP, foster relapse and worsen patient survival 102.

Senescence is a permanent irreversible state 103. Senescence-associated irreversible cell cycle exit is primarily controlled by the p53/p21 and p16INK4a/Rb pathways 104. However, either pathway can also induce a reversible cell cycle arrest, suggesting that activation of p53/p21 or p16INK4a/Rb alone does not fully account for the irreversibility of senescence-related cell cycle exit or that senescence itself is reversible. The reversibility of senescence remains controversial. One study has suggested that senescence is irreversible and that senescent cells enter and maintain this state through continuous MYC degradation 104. However, some studies have suggested that senescence is reversible 105.

Senescent cells are generally considered unrelated to tumor recurrence, mainly because of irreversible cell cycle arrest of senescent cells. However, some studies have shown that chemotherapy-induced senescent tumor cells secrete SASP-related cytokines and chemokines that further promote their tumorigenic properties 106, 107. In contrast to cells undergoing apoptosis, senescent cells do not undergo immediate death; instead, they accumulate over time following treatment 107. Therefore, regardless of whether senescent cells are reversible, eliminating senescent cancer cells to increase the chemotherapy response and prolong survival has been proposed as a potential treatment strategy.

In summary, genotoxic chemotherapeutic drugs can promote p53 signaling, leading to apoptosis, dormancy, and senescence under different conditions. Chemotherapy regimens and individual differences in patients determine the three different directions. Therefore, the role of p53 in determining cell fate should be considered when selecting chemotherapeutic strategies. However, the current research has only focused on a single aspect and lacks comprehensive studies on the relationship between apoptosis, dormancy, and senescence. Therefore, the mechanism through which chemotherapy regulates cell fate via p53 is an interesting research topic.

## 3. Stress Responses

Cellular dormancy consists of two elements: maintenance of survival and arrest of the cell cycle 108**.** The premise of awakening dormant cells is to create an environment suitable for cell growth. In response to unfavorable conditions—such as chemotherapy or the associated microenvironmental changes—dormant cancer cells can activate adaptive survival programs that allow them to persist. While some of these cells may eventually reawaken when conditions become favorable, others can remain in a dormant state indefinitely Therefore, understanding the stress response of dormant cells to chemotherapy is crucial to guide therapeutic strategies.

### 3.1 Unfolded protein response (UPR)

Chemotherapy induces endoplasmic reticulum (ER) stress and activates the UPR pathway, an adaptive survival pathway linked to chemoresistance 109. Since this stress response can facilitate cell-cycle arrest and survival of dormant cells 110, treatments that inhibit the survival mechanism of dormant cells may help eliminate dormant cells.

The UPR is a protective program that re-establishes ER homeostasis by lowering the protein burden, increasing folding capacity, and facilitating the clearance of misfolded proteins. However, when ER stress is severe or the UPR is prolonged, ER homeostasis may fail to be restored, culminating in UPR-mediated cell death. The major UPR sensors are three transmembrane proteins: protein kinase R-like ER kinase (PERK), inositol-requiring enzyme 1α (IRE1α), and activating transcription factor 6 (ATF6). During homeostasis, the ER domain of these three proteins binds to the molecular companion 78kD glucose-regulated protein (GRP78/BiP), and under ER stress, GRP78 is recruited to misfolded proteins, dissociating from the sensors and triggering their activation 111-113.

The activation of the UPR pathway induced cell cycle blockade. For example, PERK activation through ER stress inhibits cyclin D1 translation and subsequently causes arrest at the G1 phase 114. The activation of the UPR helps maintain the survival of dormant cells. In squamous carcinoma cells that are dormant because of p38 MAPK activation, the p38 MAPK signaling pathway promotes dormant cell survival by upregulating the expression of the ER chaperone BiP and enhancing PERK activation. BiP upregulation can inhibit Bax activation, which can reduce cancer cell apoptosis, thereby promoting survival 115. Nuclear factor erythroid 2-related factor 2 (Nrf2) is a transcription factor related to the regulation of cell resistance to oxidative damage 116. The acquisition of drug resistance may be related to dormancy and survival of dormant cells. Nrf2 is a key effector molecule in PERK-mediated cell survival. Activated PERK induces the Nrf2 detoxification pathway, which promotes cell survival 117. For example, constitutive PERK-Nrf2 signaling protects dedifferentiated cells from chemotherapy by reducing ROS levels 118. One study has reported that adaptation to ER stress in cancer cells produced a multidrug resistant phenotype via activation of the PERK/Nrf2/multidrug resistant-related protein 1 (MRP1) axis. PERK activates Nrf2, which binds to antioxidant response elements in the promoter region to upregulate the expression of MRP1, which promotes drug efflux and is conducive to resistance against cytotoxic chemotherapy 119.

During dormancy, p38 promotes ATF6α nuclear translocation and transcriptional activation. *In vivo,* the ATF6α-Rheb-mTOR axis selectively increases the proportion of dormant cells that survive. ATF6α downregulation induces apoptosis in dormant tumor cells and markedly prolongs survival in mice bearing dormant disease*.* Therefore, inhibiting the ATF6α-Rheb-mTOR pathway in dormant tumor cells may aid in eliminating residual disease 120. ATF6 protects theDNA damage response by activating mTOR/HSP90/BRCA1 to stabilize the expression of BRCA1 and prevent its degradation by the proteasome, which can help protect cells from ER stressors and DNA-damaging agents. Furthermore, cancer cells were sensitive to the cytotoxic effects of DOX when combined with ATF6 inhibitors 121. Therefore, when ATF6 inhibitors are used in combination with chemotherapy, they increase the cytotoxicity of chemotherapy and are beneficial for eradicating residual lesions.

The IRE1α/XBP1 signaling pathway is pivotal to chemoresistance. The IRE1α/XBP1 pathway plays an important role in CDDP-resistant ovarian cancer cells. Inhibition of IRE1α signaling resensitizes CDDP-resistant cells both* in vivo* and *in vitro* 122. This mechanism may be related to the promotion of dormancy and maintenance of dormant cell survival. The IRE1α-XBP1s can induce dormancy, and activation of IRE1α-XBP1s can increase p21 and p27 expressions, thereby triggering cell cycle arrest 123. In addition to acquiring drug resistance through dormancy, IRE1-XBP1s can promote MRP1 expression and increase drug efflux, thus conferring resistance to cytotoxic chemotherapy 124.

As mentioned earlier, whether the UPR promotes the survival or apoptosis of dormant cells depends on the intensity of the stimulus. It is reasonable to assume that the use of high-dose chemotherapy will lead to apoptosis of dormant cells; however, high-dose chemotherapy will also attack normal tissues. Therefore, whether the host can withstand it needs to be examined. Moreover, targeting the UPR should be considered. For example, a study has reported that PERK inhibitors can specifically eliminate dormant DTCs to prevent the recurrence caused by reawakening. Therefore, new treatment strategies have been proposed. PERK inhibitors can be used as adjuvant therapy to eliminate dormant minimal residual disease; thus, PERK inhibitors alone or in combination with antiproliferative therapy may result in a better outcome for patients 125.

### 3.2 Metabolism reprogramming

#### 3.2.1 Metabolic reprogramming to alleviate oxidative stress and sustain survival

As noted above, dormancy comprises two components: cell-cycle arrest and survival. In the presence of chemotherapy, dormant cells undergo a series of metabolism-related adaptations to maintain intracellular homeostasis and thereby sustain survival. For example, AMPK, an energy sensor that sustains energy homeostasis in cells. In dormant cells, intracellular ATP levels are often reduced, thereby activating AMPK 126. Accumulating evidence indicates that AMPK is involved in regulating tumor cell dormancy and can support the persistence of dormant cells. For example, metformin upregulates AMPK, inhibits mTORC1/2, and induces autophagy and G0/G1 arrest 127. By activating AMPK, metformin increases fatty acid oxidation (FAO) and mitochondrial respiration, thereby promoting the survival of dormant estrogen receptor-positive breast cancer cells 128. Despite their low-proliferative state, dormant cells must maintain adequate energy supply and intracellular homeostasis, often by shifting toward mitochondrial OXPHOS and FAO and enhancing ROS detoxification 126.

Chemotherapeutic agents increase ROS production and induce oxidative stress 129. To survive, dormant cancer cells require effective antioxidant defenses to limit oxidative stress. Under oxidative stress, AMPK functions as a central regulator of antioxidant defense by upregulating a variety of antioxidant genes 130 and activating the transcription factor Nrf2 131. A study has shown that dormant breast cancer cells adapt to oxidative stress by activating the NRF2 antioxidant pathway 132. AMPK functions as a cellular energy sensor, which suggests a close link between its metabolism and oxidative stress 133. The maintenance of redox homeostasis is closely linked to the adaptive metabolic reprogramming of dormant cells. For example, dormant DTCs can successfully reduce oxidative stress by lowering their metabolic rates via effective OXPHOS 134. In a dormant model of estrogen receptor-positive breast cancer, AMPK was upregulated, thereby increasing FAO and mitochondrial respiration 128. FAO produces numerous antioxidant molecules, such as NADPH, which help cells resist oxidative stress 135.

During chemotherapy, resistant cells typically exhibit high mitochondrial activity 136. Cisplatin-resistant cells no longer rely on glycolytic pathways, but depend on oxidative metabolism 137. Dormant cancer cells are characterized by chemoresistance and share this property as well. Experiments showed that treatment with 200 μM 5-FU markedly reduced lactate and pyruvate in dormant CRC cells, suggesting a reversal of the Warburg effect. Concurrently, metabolites in the tricarboxylic acid (TCA) cycle increased, and glutamine emerged as the primary energy source in dormant CRC cells, indicating enhanced mitochondria activity 138. Dormant cancer cells that survive after tumor genes are eliminated have the characteristics of cancer stem cells and rely on OXPHOS for survival 139. Dormant cancer cells switch from glycolysis to OXPHOS to preferentially produce more bioenergy than biomass for cell division. OXPHOS is particularly beneficial for dormant cells because these cells require energy to survive and resist multiple stressors, and OXPHOS can use alternative substrates to produce the required energy 140. FAO not only helps dormant cells resist oxidative stress but also promotes tumor cell dormancy. Nanog is a major transcription factor that maintains the self-renewal and pluripotency of stem cells. Nanog is regulated by the FAO/ATP citrate lyase-dependent pathway. FAO increases ATP citrate lyase expression and promotes acetyl-CoA synthesis Subsequently, P300 transfers acetyl-CoA and promotes the acetylation at H3K27 the Nanog promoter. Nanog upregulation increases the transcription of p21 and p27, thereby promoting dormancy of CRC cells 141. In a Her2+/Neu breast cancer model, tumor cells enter a prolonged dormant state following Her2 downregulation. Although these cells cease proliferation, they are not metabolically inactive. Instead, they undergo characteristic metabolic reprogramming during dormancy. Specifically, dormant tumor cells exhibit reduced glycolytic activity and increased mitochondrial OXPHOS, indicating a shift toward mitochondrial metabolism to sustain survival. In fast-recurrence tumors, this metabolic shift is more pronounced, with a transition from glucose utilization to FAO as the primary energy source. This adaptation appears to underlie their aggressive recurrence potential. Notably, early intervention during tumor regression and dormancy with a FAO inhibitor not only restores glucose utilization but also significantly prolongs dormancy and delays recurrence. These findings highlight the dynamic metabolic plasticity of dormant tumor cells and suggest that targeting lipid metabolism through FAO inhibition may offer a promising therapeutic window to prevent recurrence 142.

### 3.3 Autophagy

Autophagy is a lysosome-dependent intracellular degradation pathway that sequesters and degrades damaged organelles and long-lived proteins, recycling them to provide nutrients or ATP and thereby support cell survival under internal or external stress 143. Autophagy exerts a dual role following chemotherapy. On one hand, autophagy functions as a cytoprotective program that mediates acquired chemoresistance in certain cancer cells. For example, the IL-6/JAK2/BECN1 signaling pathway, which activates autophagy, contributes to CRC resistance to 5-FU and OXA 143. Positive feedback from COPS3-FOXO3 promotes the formation and maturation of autophagosomes and induces cisplatin resistance in osteosarcoma cells 144. By contrast, autophagy can also induce autophagic cell death, such as when Bax or PUMA-deficient cells (i.e., apoptosis-deficient cells) experience autophagic death after 5-FU treatment 145.

Activation of autophagy is a novel characteristic of dormant cells in diverse tumors 146.

Autophagy, activated by chemotherapeutic drugs, promotes the formation of dormant cells. For example, chemotherapy drugs (PTX [150 ng/μL] and CDDP) damage mitochondria, resulting in decreased ATP level, and activate autophagy through the AMPK-mTOR pathway, thus promoting the formation of dormant polyploid giant cancer cells in nasopharyngeal carcinoma 147. Autophagy maintains dormant cell survival. Autophagy inhibition leads to apoptosis in dormant cells 148. Therefore, autophagy participates in the induction and maintenance of cancer cell dormancy and helps cells cope with stress. Therefore, inhibition of autophagy is a promising treatment. Inhibiting autophagy significantly reduces the ability of dormant breast cancer cells to survive *in vivo* and *in vitro*. However, when these dormant cells enter a proliferative state, the inhibition of autophagy has little effect on tumor metastasis, suggesting the important role of autophagy in the early stages of dormancy. Therefore, the timing of autophagy inhibitor use is crucial 149.

Mitophagy selectively clears damaged mitochondria through lysosomal turnover 150.

Chemotherapy induces mitochondrial autophagy and promotes tumor cell dormancy. Mitochondrial autophagy levels are elevated in head and neck squamous cell carcinoma cell lines treated with CDDP (1/10 of the IC50* in vitro*; 4 mg/kg *in vivo*), leading to a dormant state in tumor cells 151. Mitochondria-induced autophagy plays an important role in the dormant cells exposed to chemotherapy-induced oxidative stress. Damaged mitochondria both fail to generate ATP and release higher ROS levels 152. Therefore, the timely removal of damaged mitochondria has become a key strategy to deal with oxidative stress. Mitochondrial autophagy also prevents oxidative stress-induced damage. ROS cause DNA damage. In dormant breast cancer cells, inhibition of mitochondrial autophagy leads to elevated γH2Ax and cCASP3 levels, indicating cell damage 149.

### 3.4 Prospects

Owing to the influence of chemotherapy on tumor metabolism, metabolic changes are non-negligible causes of tumor dormancy and drug resistance during treatment. Adjuvant drugs that disable survival metabolism in dormant cells will increase the effectiveness of chemotherapy.

## 4. Discussion

Chemotherapy is widely used as a standard treatment for cancer treatment. However, chemotherapy has dose- and schedule-dependent effects: it can push tumor cells into dormancy or, conversely, reawaken quiescent cells. Therefore, when designing therapeutic strategies, it is essential to fully consider how chemotherapeutic agents regulate cell dormancy, as this phenomenon can have profound implications for treatment outcomes. On one hand, maintaining dormancy may help delay disease recurrence and improve patients' quality of life; on the other hand, dormant cells have a potential risk of sudden reactivation and metastatic spread. In contrast, in some cases, treatment strategies that attempt to awaken dormant cells for subsequent elimination may paradoxically accelerate disease progression 153. Thus, mechanistic insight into dormancy-regulating is pivotal to determining whether maintaining or eliminating dormant cells is more appropriate in specific clinical settings.

Although research on the mechanisms of cell dormancy has advanced, detecting and definitively identifying the dormant state remains difficult. First, the lack of uniform and highly specific biomarkers makes it difficult to accurately identify and dynamically monitor dormant cells in clinical practice. This significantly limits early assessment of recurrence risk and the implementation of targeted therapies. Second, technical bottlenecks remain in the isolation and detection of circulating tumor cells and DTCs. Moreover, dormancy itself is highly heterogeneous, and a single marker is insufficient to comprehensively define or predict the fate of dormant cells. To make matters more complex, significant discrepancies exist between in vitro models and in vivo dormant states, further complicating detection and intervention efforts 154.

**Figure 2:**
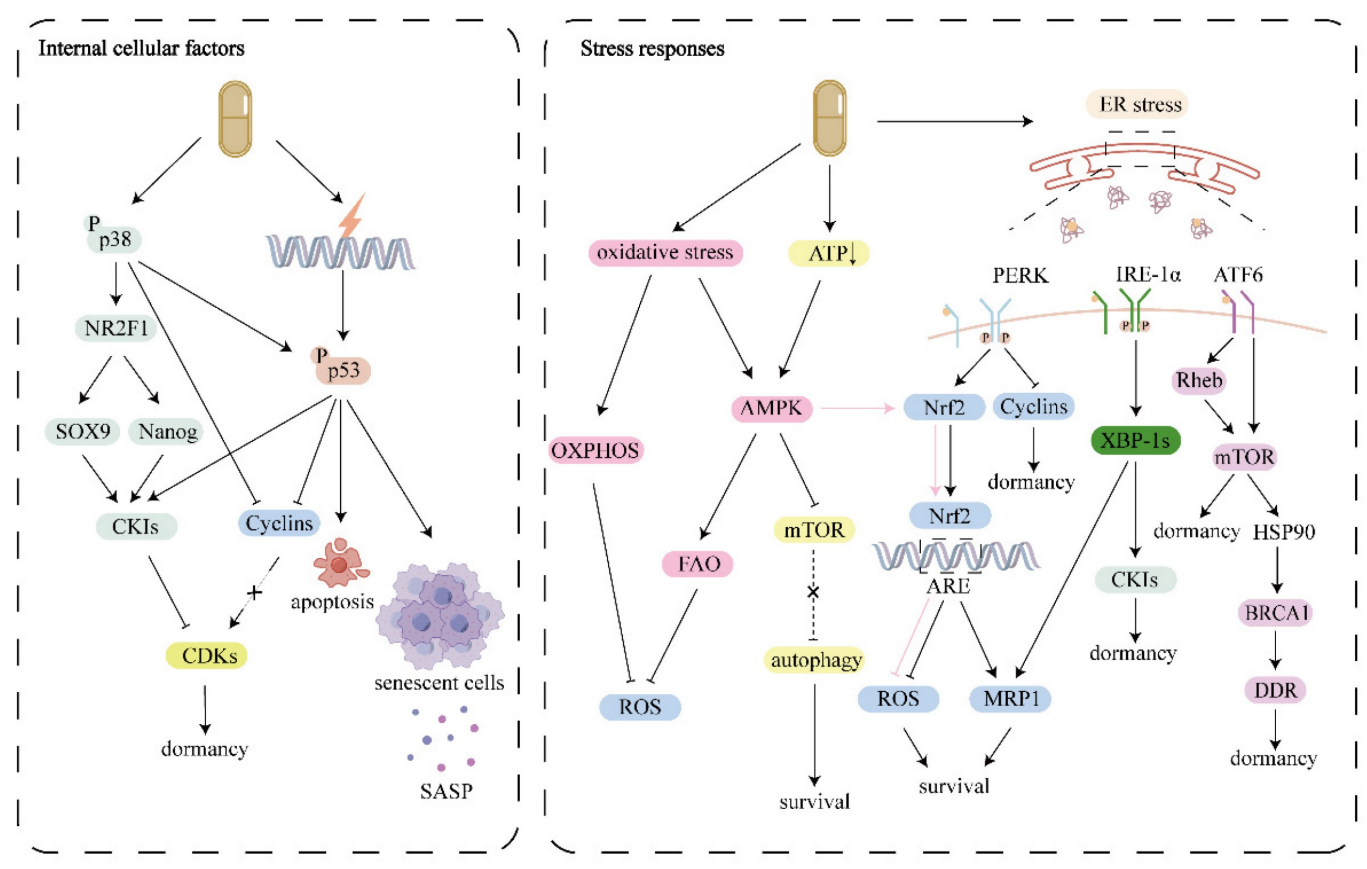
Internal cellular factors: Chemotherapy activates stress activated p38 MAPK and p53, which regulate the cell cycle by inhibiting cyclins and promoting CKIs, ultimately suppressing CDKs and inducing cells into dormancy. Stress responses: Chemotherapy can trigger survival stress including ER stress, oxidative stress and energy deficiency. In order to cope with these survival pressures, dormant cells activate UPR, antioxidant response and metabolic reprogramming to promote cell survival. TME, tumor microenvironment; CKI, CDK inhibitors; ER, endoplasmic reticulum; ECM, extracellular matrix; UPR, unfolded protein response.

The threshold for the transition from cytotoxic to dormant induction of chemotherapy is also undetermined. Future studies should focus on elucidating the mechanisms underlying these transitions, validating dormancy-associated targets, and exploring personalized adaptive therapeutic strategies that balance tumor suppression with recurrence prevention (**Fig. 2**).

## Abbreviations

5-FU: 5-fluorouracil
AMPK: AMP-activated protein kinase
ATF6: activating transcription factor 6
CAF: cancer-associated fibroblast
CDDP: cisplatin
CDK: cyclin-dependent kinase
CKI: CDK inhibitors
CRC: colorectal cancer
DOX: doxorubicin
ECM: extracellular matrix
ER: endoplasmic reticulum
ERK: extracellular signal-regulated kinase
IFN: interferon
IRE1α: inositol-requiring enzyme 1α
MDSC: myeloid-derived suppressor cell
MRD: minimal residual disease
MRP1: multidrug resistant-related protein 1
MTD: maximum tolerated dose
NETs: neutrophil extracellular traps
NK: natural killer
NR2F1: nuclear receptor subfamily 2 group F member 1 gene
Nrf2: nuclear factor erythroid 2-related factor 2
OXA: oxaliplatin
OXPHOS: oxidative phosphorylation
PERK: protein kinase R-like ER kinase
PTX: paclitaxel
ROS: reactive oxygen species
SASP: senescence-associated secretory phenotype
TME: tumor microenvironment
Tregs: regulatory T cells
UPR: unfolded protein response
VEGF: vascular endothelial growth factor
